# The Gut Microbiota of an Individual Varies With Intercontinental Four-Month Stay Between Italy and Nigeria: A Pilot Study

**DOI:** 10.3389/fcimb.2021.725769

**Published:** 2021-11-22

**Authors:** Ayorinde O. Afolayan, Elena Biagi, Simone Rampelli, Marco Candela, Patrizia Brigidi, Silvia Turroni, Funmilola A. Ayeni

**Affiliations:** ^1^ Department of Pharmaceutical Microbiology, Faculty of Pharmacy, University of Ibadan, Ibadan, Nigeria; ^2^ Unit of Microbiome Science and Biotechnology, Department of Pharmacy and Biotechnology, University of Bologna, Bologna, Italy; ^3^ Department of Medical and Surgical Sciences, University of Bologna, Bologna, Italy; ^4^ Department of Biology, Simmons University, Boston, MA, United States

**Keywords:** gut microbiota, plasticity, 16S rRNA gene sequencing, inferred metagenomics, international travel, short stay, Nigeria, Italy

## Abstract

Despite well-established knowledge of the role of diet and the geographic effect on the gut microbiota of human populations, the temporal dynamics of the individual microbiota profile across changes associated with intercontinental short residence are still far from being understood. This pilot study sought to provide insights into the trajectory of the gut microbiota of an individual during a two-month stay in Italy and a subsequent two-month stay in Nigeria, by 16S rRNA gene sequencing and inferred metagenomics. The gut microbiota underwent massive but temporary changes, both taxonomically and based on predicted functionality. The faecal microbiota associated with the short stay in Italy progressively lost diversity and showed a dominance of Firmicutes, while after returning to Nigeria, the microbial community quickly regained the typical profile, in terms of biodiversity and bacterial signatures of traditional lifestyle, i.e., *Prevotella* and *Treponema*. Predicted pathways involved in glycolysis, fermentation and N-acetylneuraminate degradation were enriched during the subsequent two-month stay in Nigeria, whereas pathways associated with amino acid and peptidoglycan synthesis and maturation became over-represented during short stay in Italy. Our findings stress the plasticity of the individual gut microbiota even during a short-term travel, with loss/gain of taxonomic and functional features that mirror those of the gut microbiota of indigenous people dwelling therein.

## Introduction

Every human on Earth harbours a uniquely diverse community of microorganisms in the gut. The community’s structure and functions are driven by endogenous and exogenous factors, including diet, lifestyle, xenobiotics exposure, geographical location, ethnicity and minimally host genetics (Li et al., 2016; Zhernakova et al., 2016; Deschasaux et al., 2018; He et al., 2018). Diet and geographical location, in particular, constitute the major factors influencing the gut microbiota variation (Nakayama et al., 2017; Turnbaugh, 2017; He et al., 2018; Vangay et al., 2018). It is recognized that there is a shifting trend in the taxonomic and functional profile of the gut microbiota as individuals adhering to traditional (i.e., foraging and agricultural) subsistence strategies adjust to industrialization and urbanization, adopting a Westernized lifestyle (Ayeni et al., 2018; Afolayan et al., 2019). Furthermore, such a shifting trend has been shown to be associated with the migration of individuals from non-Westernized to Westernized countries (Vangay et al., 2018). These shifts include (but are not limited to): loss of gut microbiota diversity and loss of traditional lifestyle-associated microbial taxa (the so-called VANISH taxa) and functions (Sonnenburg and Sonnenburg, 2019a; Sonnenburg and Sonnenburg, 2019b). These events may have negative implications for public health, as evidenced by the decrease in the ‘healthiness’ of the gut microbiota and by the increased risk of obesity and other chronic diseases in immigrants (Kootte et al., 2012; Martínez et al., 2015; Vangay et al., 2018). Although evidence on the influence of migration on the gut microbiota exists, a complete understanding of the gut microbiota dynamics associated with an international travel and short stay in a Westernized nation and subsequently a non-Western one is currently lacking.

In an attempt to shed light in this direction, here we conducted a pilot study to determine whether a short-term change in the geographical location between a Western (Italy) and non-Western (Nigeria) nation could result in changes in the structure and functionality of the intestinal microbiota, mirroring the microbial profile of populations residing in the respective countries.

## Materials and Methods

### Faecal Sampling and Collection of Diet and Lifestyle Information

The volunteer is a middle-class woman living an average middle-class life in Nigeria and Italy during the four-month period of sample collection. There was no change in her lifestyle during the stay in Nigeria and Italy. Drugs including antibiotics were not used by the volunteer during the period of sample collection in either country. The volunteer lived in urban environment in Nigeria and Italy, and was working during her stay in both countries. Besides the consumption of pizza sporadically during the stay in Italy, similar foods were consumed by the volunteer in Nigeria and Italy, e.g., fruit, rice, beans, animal proteins (chicken, beef, fish), spicy pepper, ice cream, chocolate, and vegetables. However, the sources of the foods (Italy *versus* Nigeria) were obviously different according to the geographical locations. The foods consumed by the volunteer in both countries were obtained from local grocery stores and most of the foods were industrially processed.

As shown in **Supplementary Table 1**, faecal samples were collected from the volunteer while on a two-month short stay in Italy from the 1st week until the 8th week, starting on the 3rd day after arrival in Italy (Bologna, Italy). On return to Nigeria, faecal samples were collected from the 1st week until the 9th week, starting on the 3rd day after arrival into Nigeria. Sampling was carried out once a week, except for the 2nd week in Italy, which involved two samplings. All samples were processed in Bologna (Italy) for DNA extraction and Illumina sequencing as previously described (Ayeni et al., 2018) and detailed below.

### Microbial DNA Extraction and 16S rRNA Gene-Based Illumina Sequencing

The bead beating-column combination method (Yu and Morrison, 2004) was employed in the extraction of microbial DNA from faeces, with slight modifications (Biagi et al., 2016). Briefly, faeces were suspended in tubes containing four 3-mm glass beads, 0.5 g of 0.1-mm zirconia beads and 1 mL of lysis buffer [500 mM NaCl, 50 mM Tris-HCl, 50 mM EDTA, 4% (w/v) SDS], and were treated thrice in a FastPrep instrument (MP Biomedicals, Irvine, CA, USA) at a speed of 5 movement/s within a 1-minute period. This was followed by a 15-minute incubation at 95°C, centrifugation in order to pellet particles of stool, and precipitation of nucleic acids by the addition of 10 M ammonium acetate and 1 volume of isopropanol. Ethanol (70%) was employed for washing the pellets, followed by resuspension in 10 mM Tris-HCl, 1 mM EDTA pH 8.0 (TE) buffer. The suspension was treated with DNase-free RNase (10 mg/mL) at 37°C for 15 minutes, protein was removed, and the DNA was purified according to the manufacturer’s protocol (QIAamp DNA Stool Mini Kit; QIAGEN, Hilden, Germany).

The primers 341F and 805R (with added Illumina adaptor sequences) were used for the targeted amplification of the V3-V4 region of the 16S rRNA gene as described previously (Candela et al., 2016). A magnetic bead-based clean-up system (Agencourt AMPure XP; Beckman Coulter, Brea, CA, USA) was used to purify the amplicons. The Nextera technology was used to prepare indexed libraries by limited-cycle PCR. After further purification and pooling at equimolar concentrations, the final library was denatured with 0.2 N NaOH and diluted to 6 pM with a 20% PhiX control. This was followed by paired-end sequencing (2 × 300 bp) on Illumina MiSeq platform.

### Bioinformatics and Statistical Analysis of Sequence Data

The Illumina amplicon sequence data were analysed with the use of the QIIME 2 pipeline (qiime2-2019.7) on a local machine, according to the procedure outlined by the QIIME team (Bolyen et al., 2018). Briefly, paired-end reads were imported (qiime tools import) into a QIIME 2 artifact and merged for further processing. Demultiplexed paired-end reads were visualized (qiime tools view) in order to check the quality of the sequence reads. This procedure informed the choice of the sequence positions to trim off during the quality control step. Quality control of the sequences (including filtering, denoising, and chimera removal) was achieved with the use of DADA2 pipeline (Callahan et al., 2016) incorporated into QIIME 2 (qiime dada2 denoise-paired). This was followed by the generation of a tree for phylogenetic diversity analyses (qiime phylogeny align-to-tree-mafft-fasttree). Taxonomy was assigned to the remaining high-quality sequence reads using the q2-feature-classifier plugin trained on the Greengenes 13_8 reference database (DeSantis et al., 2006).

Alpha rarefaction was determined by selecting a maximum sampling depth of 20,000 (qiime diversity alpha-rarefaction). Alpha diversity and beta diversity were assessed with the use of the q2 diversity plugin, as well as the R packages tidyverse (v1.3.0) (Wickham et al., 2019), ggpubr (v0.4.0) (Kassambara, 2020), phyloseq (v1.26.1) (McMurdie and Holmes, 2013), and qiime2R (v0.99.4) (https://github.com/jbisanz/qiime2R/). Data were visualized using ggplot2 (v3.3.3) (Wickham, 2009). The metrics used for alpha diversity included Faith’s phylogenetic diversity, observed ASVs, Shannon index, inverse Simpson and Pielou’s evenness index. Wilcoxon rank sum test was used to assess differences in alpha diversity between Italy and Nigeria stay.

Unweighted and weighted UniFrac distances, as well as Bray-Curtis and Jaccard distances were used as measures of beta diversity. Beta diversity distances were used as input for the generation of Principal Coordinate Analysis (PCoA) plots, and the separation within the plots was assessed for significance by PERMANOVA, using 999 Monte Carlo Permutations. Associations between metadata (i.e., geographical location) and alpha and beta diversity were determined (qiime diversity alpha-group-significance, and qiime diversity beta-group-significance, respectively). The feature table was exported to LEfSe on the Galaxy platform maintained by the Huttenhower research group (https://huttenhower.sph.harvard.edu/galaxy/) for discriminant taxa analysis (Segata et al., 2011). LDA score >4 was chosen as the threshold. Taxonomic barplots were constructed using the R packages phyloseq (v1.26.1) and ggplot2 (v3.3.3). The functional profiles of the microbial communities were inferred by using the picrust2 plugin on QIIME 2 (qiime picrust2 full-pipeline) (Douglas et al., 2019). Functional alpha and beta diversity were measured using the same standard commands/plugins implemented for taxonomic diversity on the QIIME 2 pipeline. Discriminant functional pathways for Italian and Nigerian stays were identified by using the LEfSe function, with an LDA score >3 as the threshold.

### Limitation of the Study

Due to technical issues, the baseline sample before the volunteer left Nigeria was not included in this study. The first sampling was on arrival in Italy.

## Results

**Figure 1:**
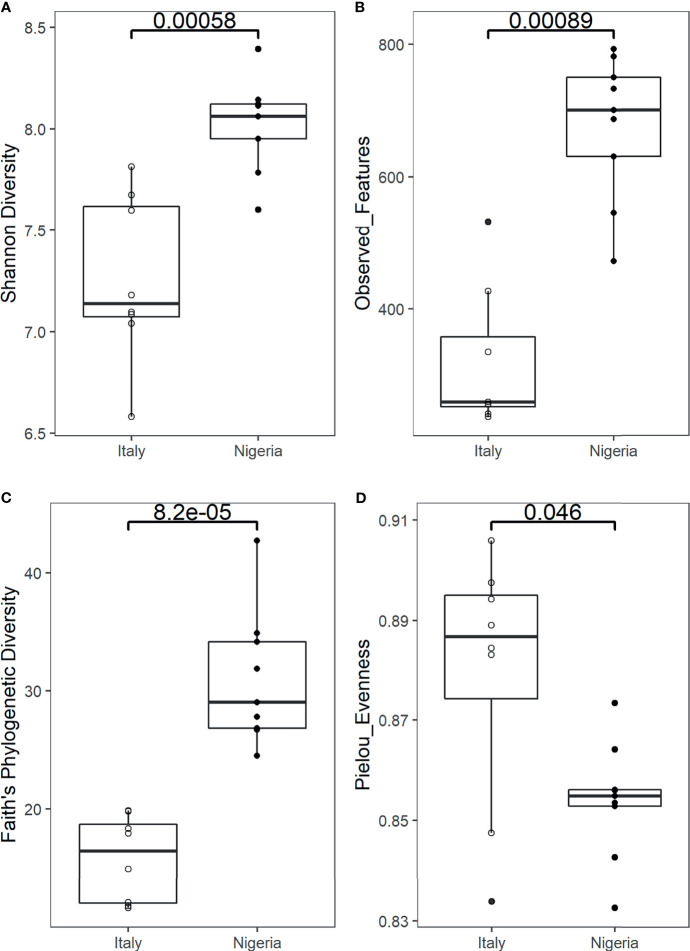
Alpha diversity of an individual’s gut microbiota during a 2-month stay in Italy, and back to Nigeria. Boxplots show a significantly higher Shannon diversity **(A)**, number of observed ASVs **(B)** and Faith’s phylogenetic diversity **(C)**, while a significantly lower Pielou’s evenness index **(D)** in the faecal microbiota of samples collected from Nigeria (n=9) compared to Italy (n=9) (p = 0.00058, p = 0.00089, p = 0.000082 and p = 0.046, respectively). Statistical test: Wilcoxon rank sum test.

Faecal samples were obtained weekly from an individual while on a two-month short stay in Italy (n=9) and upon returning to Nigeria for two months (n=9) (**Supplementary Table 1**). Bioinformatic analysis of 16S rRNA gene sequence data derived from Illumina sequencing yielded 1,299,262 high-quality reads with an average of 72,181 ± 37,653 reads per sample. Reads were clustered into 4,376 amplicon sequence variants (ASVs). Reads were rarefied to a sequencing depth of 20,000 (**Supplementary Figure 1**). According to several metrics, the alpha diversity of the gut microbial community during stay in Italy is significantly lower than that of samples collected after returning to Nigeria (Faith’s phylogenetic diversity, p = 0.000082; number of observed ASVs, p = 0.00089; Shannon index, p = 0.00058; Wilcoxon rank sum test) (**Figures 1A–C**). When focusing on evenness, an opposite trend consisting of lower values detected shortly after arrival in Nigeria was recorded (Pielou’s evenness index, p = 0.046) (**Figure 1D**). On a weekly scale, the greatest variation was observed during the stay in Italy, with a progressive decrease in richness down to the lowest value of Shannon index on week 4, while upon returning to Nigeria the diversity values were found to be generally more stable (**Supplementary Figure 2**).

**Figure 2:**
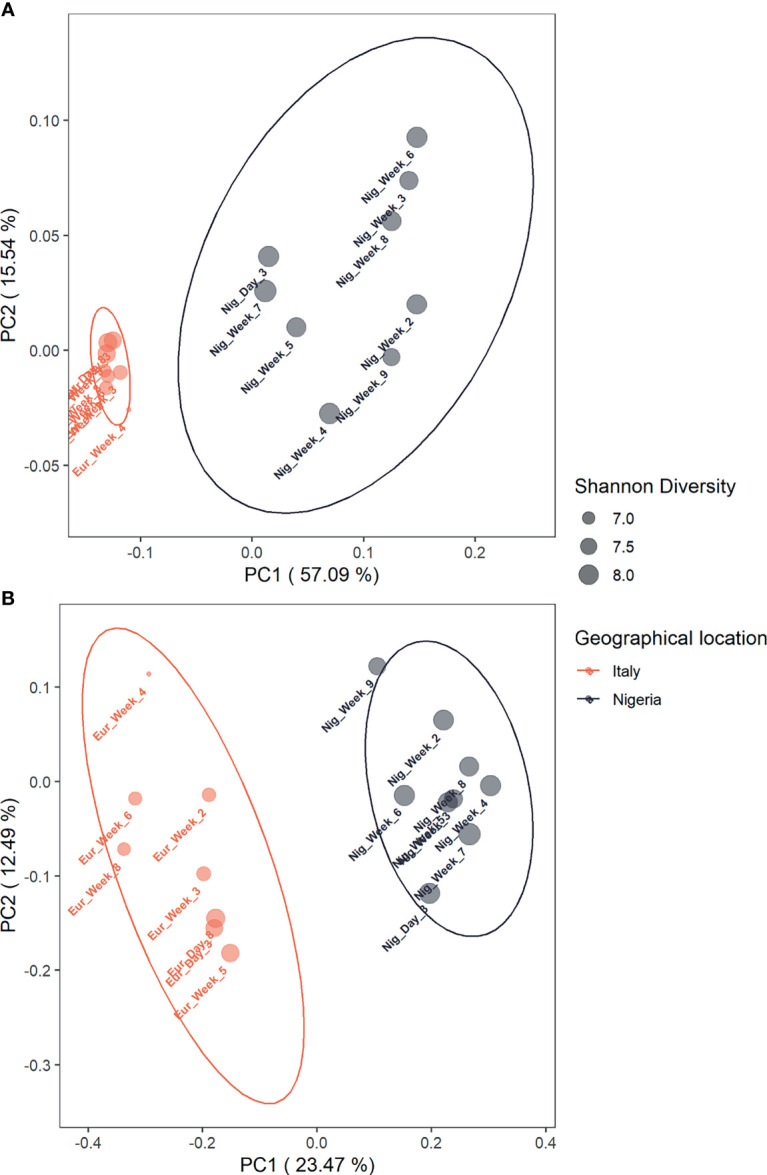
The compositional structure of an individual’s gut microbiota while in Italy segregates from the profile associated with life in Nigeria. Principal Coordinates Analysis based on weighted **(A)** and unweighted **(B)** UniFrac distances between the Italian and Nigerian gut microbiota profiles shows significant segregation (p = 0.001; PERMANOVA). Samples were labelled by day or week of collection (see **Supplementary Table 1**) and coloured by geographical location. Ellipses include 95% confidence area based on the SE of the weighted average of sample coordinates and are colored by geographical location (Italy: red, Nigeria: black). Dot size is proportional to the Shannon index, as shown in the legend.

As for beta diversity, the gut microbiota profiles related to stays in Italy and Nigeria are significantly separated from each other, based on weighted and unweighted UniFrac distances (p = 0.001; PERMANOVA) (**Figures 2A, B**), Bray-Curtis and Jaccard distances (p = 0.001) (**Supplementary Figure 3**). It should also be noted that in the weighted UniFrac-based PCoA, lower inter-sample variation was observed for the gut microbiota structures while on short stay in Italy.

**Figure 3:**
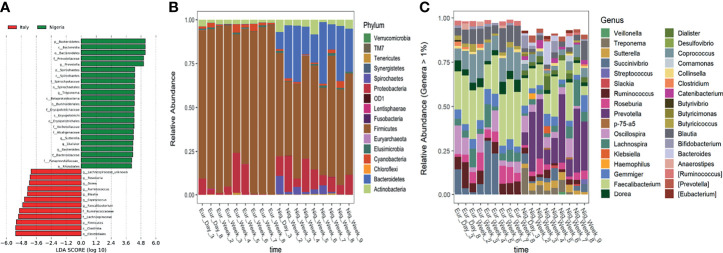
Discriminant taxa between Italy and Nigerian stay. **(A)** Differentially represented taxa were identified by linear discriminant analysis (LDA) effect size (LEfSe) analysis. The logarithmic threshold for discriminative features was set to 4.0. Bar plots showing the phylum- **(B)** and genus-level **(C)** composition of microbial communities in faecal samples collected during the stay in Italy and Nigeria.

Based on a LEfSe analysis, several country-discriminant taxa were identified (**Figure 3A**). For instance, Firmicutes largely dominated all faecal samples during the short stay in Italy while a significant enrichment of members of the phyla Bacteroidetes, Spirochaetes and Actinobacteria was observed from the first day after returning to Nigeria (**Figure 3B**). At the genus level, the typical signatures of a Western-like microbiota, i.e., *Coprococcus*, *Faecalibacterium*, *Blautia*, *Ruminococcus*, *Dorea* and *Roseburia*, were significantly enriched during the short stay in Italy, while the microbial signatures of traditional lifestyle, such as *Prevotella*, *Treponema*, *Sutterella* and *Dialister* were significantly enriched after returning to Nigeria (**Figure 3C**).

**Figure 4:**
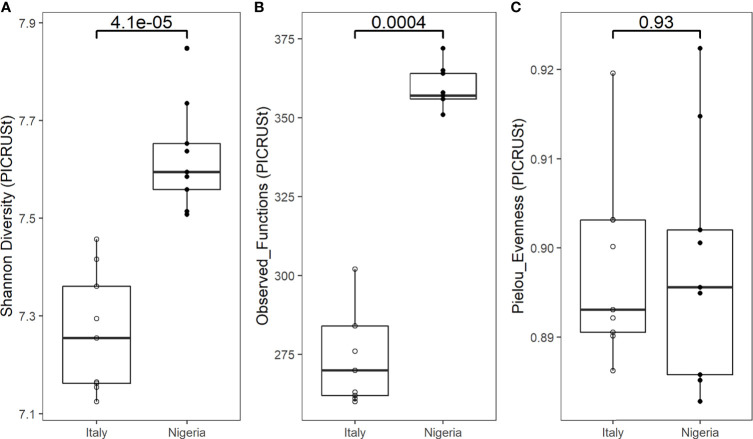
Functional alpha diversity of an individual’s gut microbiota during the stay in Italy, and back to Nigeria. Boxplots show a significantly higher Shannon diversity **(A)** and number of observed functions **(B)** in the faecal microbiota of samples collected from Nigeria (n=9) compared to Italy (n=9) (p = 0.000041 and p = 0.00044, respectively). No difference in Pielou’s evenness **(C)** was observed (p = 0.93). Statistical test: Wilcoxon rank sum test.

Indeed, the PICRUSt-predicted gut metagenome of the individual during the Italy short stay is less diverse (p < 0.0005, Wilcoxon rank sum test) (**Figures 4A, B**) and functionally different (p = 0.001, PERMANOVA) (**Supplementary Figure 4**) than that of the same individual a few days after returning to Nigeria. No difference was found in evenness (p = 0.93, Wilcoxon rank sum test) (**Figure 4C**).

Predicted pathways enriched in the gut while in Italy include pathways involved in peptidoglycan synthesis and maturation, glycogen biosynthesis and degradation, biosynthesis of amino acids (including isoleucine, glutamine, valine, serine, and glycine), and degradation of simple and complex sugars (sucrose and starch) (**Supplementary Figure 5**). On the other hand, pathways involved in the conversion of sugars and N-acetylneuraminate to various by-products (ATP, pyruvate, lactate, ethanol, and acetate) were enriched in the gut after the woman’s return to Nigeria (**Supplementary Figure 5**).

## Discussion

This pilot study shows the gut microbiota dynamics of a Nigerian individual on a two-month short stay in Italy and after returning to Nigeria for two months. Specifically, during the short stay in Italy, along with a loss of biodiversity, the composition of the gut microbiota immediately shifts loosing traditional lifestyle-associated microbial taxa [i.e., members of Bacteroidetes (*Prevotella*) and Spirochaetes (*Treponema*)] and acquiring taxa typical of Westernized microbiotas [Firmicutes (*Blautia*, *Faecalibacterium*)], supporting previous observations on the long-term dynamics of gut microbiota in US immigrants, undergoing to progressive microbiome Westernization already after few months upon their arrival (Schnorr et al., 2014; Ayeni et al., 2018; Vangay et al., 2018; Afolayan et al., 2019). Furthermore, predicted functional pathways unique to traditional lifestyles, including the catabolism of polysaccharides (Liu et al., 2016; Ayeni et al., 2018) quickly became deficient in the individual’s gut during the short stay in Italy, while pathways involved in the degradation of simple sugars (i.e., sucrose degradation) and biosynthesis of amino acids get rich quickly. This is consistent with the metabolomics data available for traditional African and urban Italian populations, with the latter showing a remarkable enrichment of amino acids and derivatives in faeces (Turroni et al., 2016). It should also be noted that, after returning to Nigeria, we found an overrepresentation of pathways involved in the degradation of N-acetylneuraminate, a sialic acid commonly found in mucus associated with the gastrointestinal tract. This could be related to the significant rise in the relative abundance of Spirochaetes, Bacteroidetes and Enterobacteriales members, many of which are known to possess neuraminidase/sialidase activity and to use removed sialic acid as a nutrient source and for host immune system evasion (Kurniyati et al., 2013; Haines-menges et al., 2015; Han et al., 2021).

There is limited data on the aforementioned gut microbiota dynamic patterns, as it is rarely reported that on migrating to industrialized regions, dietary and lifestyle changes are associated with gut microbiota alterations, including loss of diversity and variations in specific taxa and functions (Vangay et al., 2018). Experiments in mice have shown that the effects induced by Western diets low in microbiota-accessible carbohydrates are no longer recoverable over multiple generations, except with targeted microbiota reprogramming strategies (Sonnenburg et al., 2016). As recently discussed, such alterations, if prolonged over time, could lead to the definitive loss of our ancestral microbial heritage, with the outbreak of the ‘microbiota insufficiency syndrome’, and contribute to explaining the ever-increasing emergence of gastrointestinal and extra-intestinal disorders (Sonnenburg and Sonnenburg, 2019a). However, our data highlight a relevant level of resilience of the human gut microbiome in the short term, being capable of temporary shifts toward a Western-like configuration during a short-stay in Italy and reacquiring the traditional layout immediately after the return to Nigeria. It should be noted that due to a technical issue, it was not possible to collect a faecal sample before leaving Nigeria. While we are perfectly aware that this suboptimal sampling design prevented us from having a real baseline before the Italian stay, considering the rapid microbiota shift experienced upon returning to Nigeria, it is reasonable to assume that the Nigerian configuration may approximate the typical basal profile of the volunteer. Regardless of this, our study still allowed us to determine whether a change in the geographical location could lead to changes in the structure and inferred functionality of the gut microbiota, by reconstructing in greater detail what happens in the international travel between Italy and Nigeria.

In summary, our findings demonstrate the plasticity and adaptability of the gut microbiota in a Nigerian individual to international travel and short stay. Knowledge on gut microbiota dynamics associated with international travel-related changes within each individual is pertinent to understanding the role of the gut microbiota in the onset and progression of diseases, which is of immense importance in national and global public health, particularly in terms of a better understanding of the degree of resilience with respect to an environmental stressor. Further studies in larger cohorts of subjects possibly making the same type of international travel under exposure conditions that are as comparable as possible are needed to validate these findings and extend their generalizability.

## Data Availability Statement

The datasets presented in this study can be found in online repositories. The names of the repository/repositories and accession number(s) can be found below: https://www.ncbi.nlm.nih.gov/, SAMN14451445 - SAMN14451463.

## Ethics Statement

The study was conducted according to the guidelines of the Declaration of Helsinki, and approved by the Institutional Review Board (or Ethics Committee) of the Institute of Advanced Medical Research and Training (IAMRAT), College of Medicine, University of Ibadan, Ibadan, Nigeria (UI/EC/15/0050). 

## Author Contributions

Conceptualization, FAA, EB, MC, SR, PB, and ST; Methodology, AOA, FAA, EB, SR, and ST; Formal analysis, AOA, ST, and FAA; Investigation, EB, FAA, and ST; Resources, FAA, EB, ST, and MC; Data curation, AOA; Writing—original draft preparation, AOA; writing—review and editing, FAA and ST; Visualization, AOA, FAA, and ST; Project administration, FAA and ST. All authors have read and agreed to the published version of the manuscript.

## Conflict of Interest

The authors declare that the research was conducted in the absence of any commercial or financial relationships that could be construed as a potential conflict of interest.

## Publisher’s Note

All claims expressed in this article are solely those of the authors and do not necessarily represent those of their affiliated organizations, or those of the publisher, the editors and the reviewers. Any product that may be evaluated in this article, or claim that may be made by its manufacturer, is not guaranteed or endorsed by the publisher.
