# Supplementary material for: The Gut Microbiota of an Individual Varies With Intercontinental Four-Month Stay Between Italy and Nigeria: A Pilot Study
Source: Front Cell Infect Microbiol. 2021 Nov 22;11:725769. doi: 10.3389/fcimb.2021.725769 (PMC8646098; doi:10.3389/fcimb.2021.725769)
Supplement: Supplementary file 1 [file DataSheet_1.docx]

Supplementary Material


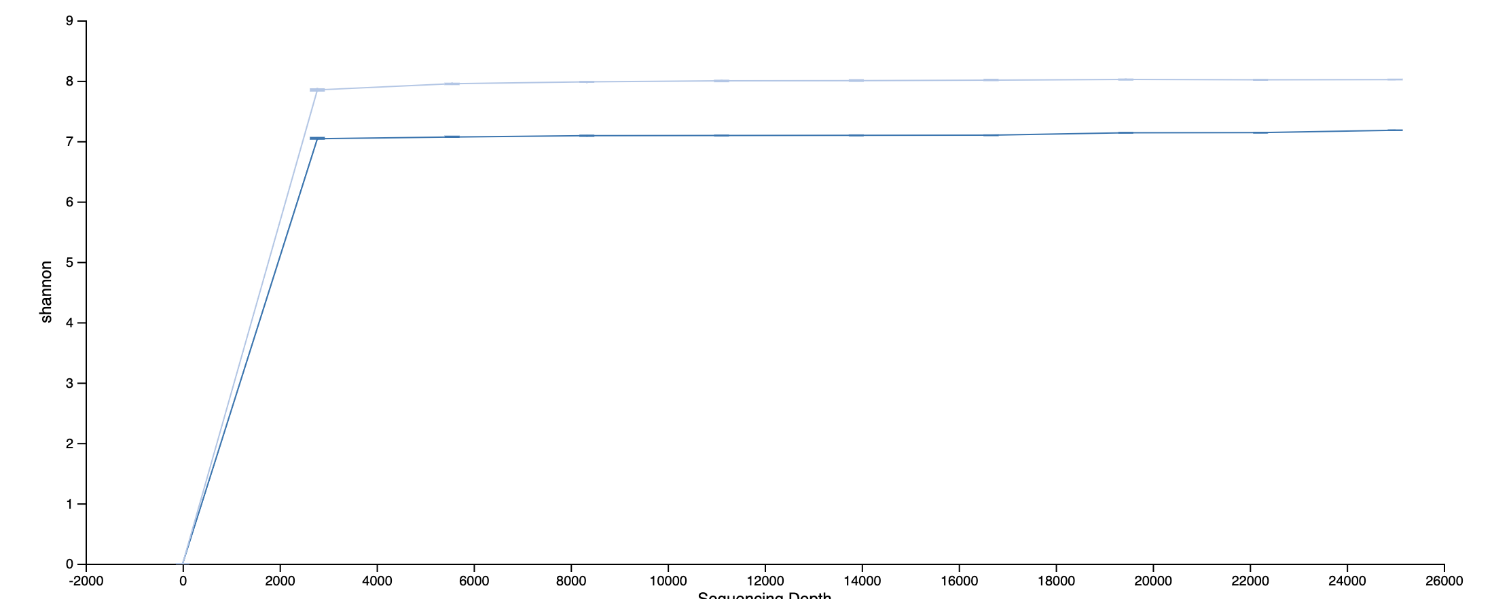


Supplementary Figure 1: Sequence data was rarefied to a sequence depth of 20,000. Dark blue, faecal samples collected during short stay in Italy; light blue, faecal samples collected after returning to Nigeria.


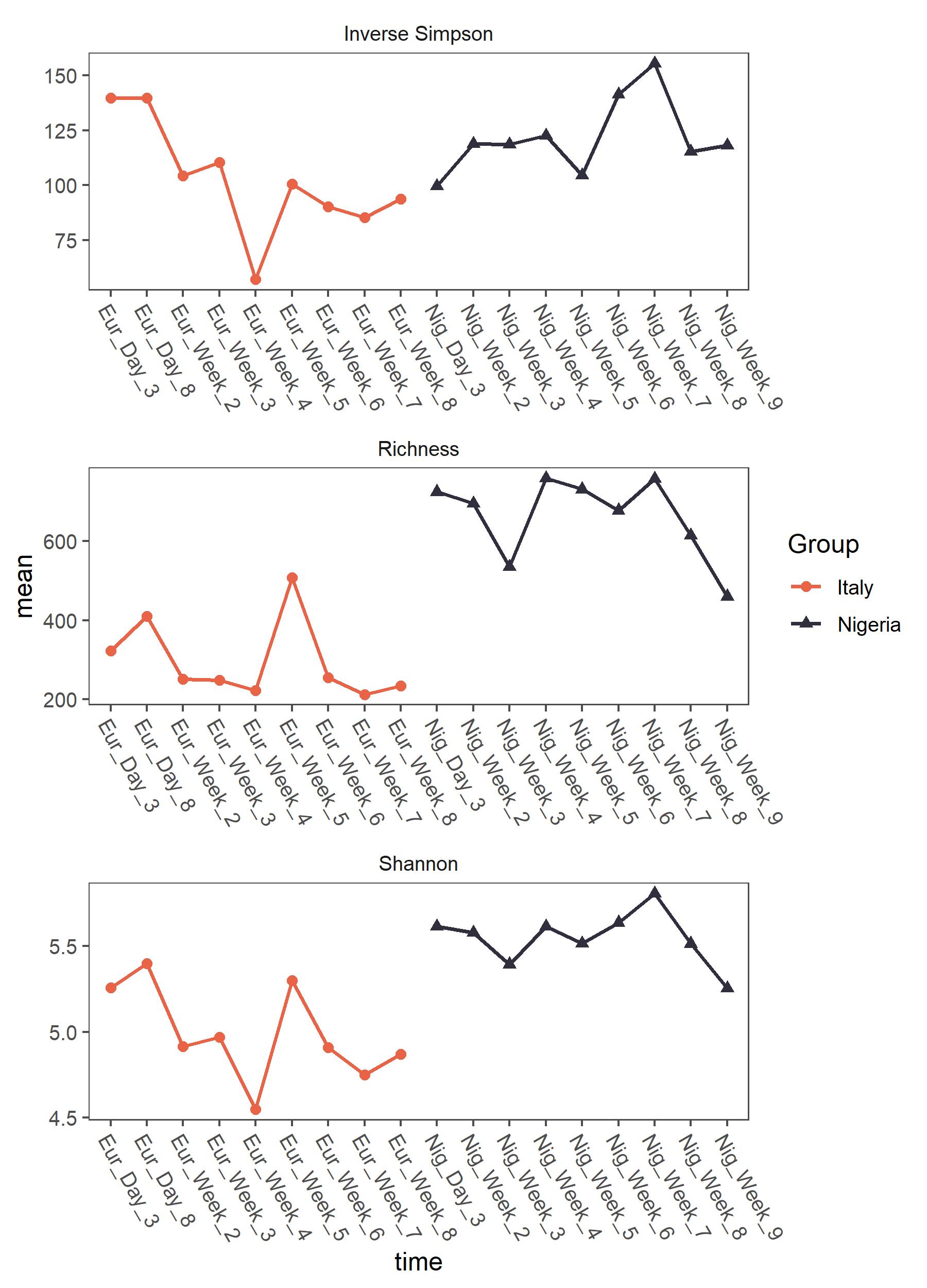


Supplementary Figure 2: Alpha diversity measures in a timeseries.

Daily dynamics of microbiota diversity according to Inverse Simpson (top), Richness (middle), and Shannon Index (bottom) from an individual on a short stay in Italy (3^rd^ day until the 8^th^ week of sample collection in Italy, Eur_Day_3 - Eur_Week_8) compared to Nigeria (3^rd^ day until the 9^th^ week of sample collection in Nigeria: Nig_Day_3 - Nig_Week_9). See also Supplementary Table 1.


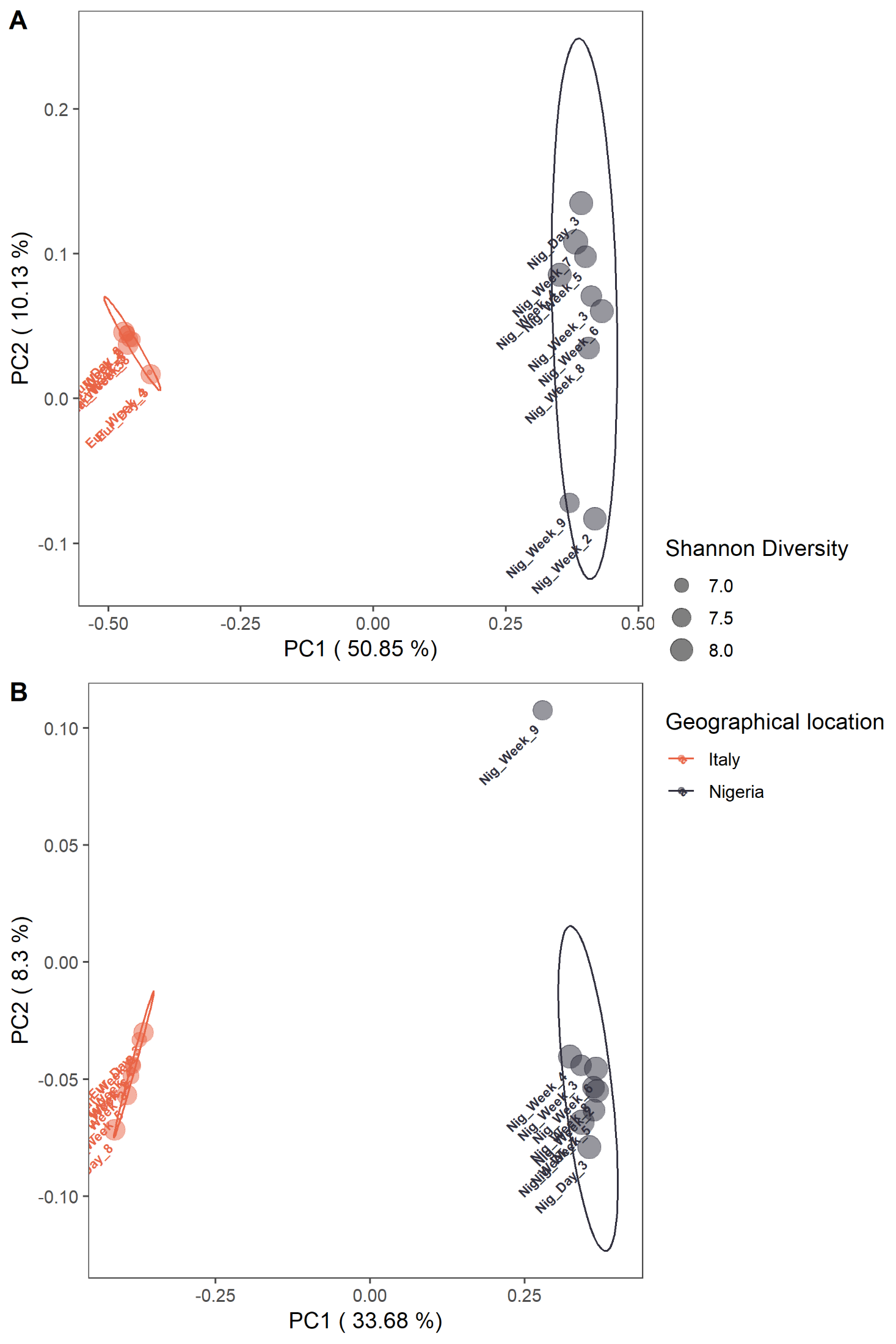


Supplementary Figure 3: The compositional structure of an individual’s gut microbiota while in Italy segregates from the microbiota profile associated with life in Nigeria. Principal Coordinates Analysis based on Bray-Curtis (A) and Jaccard (B) distances between the Italian and Nigerian gut microbiota profiles shows significant segregation (p = 0.001; PERMANOVA). Samples were labelled by day or week of collection (see Supplementary Table 1) and coloured by geographical location. Ellipses include 95% confidence area based on the SE of the weighted average of sample coordinates and are coloured by geographical location (Italy: red, Nigeria: black). Dot size is proportional to the Shannon index, as shown in the legend.


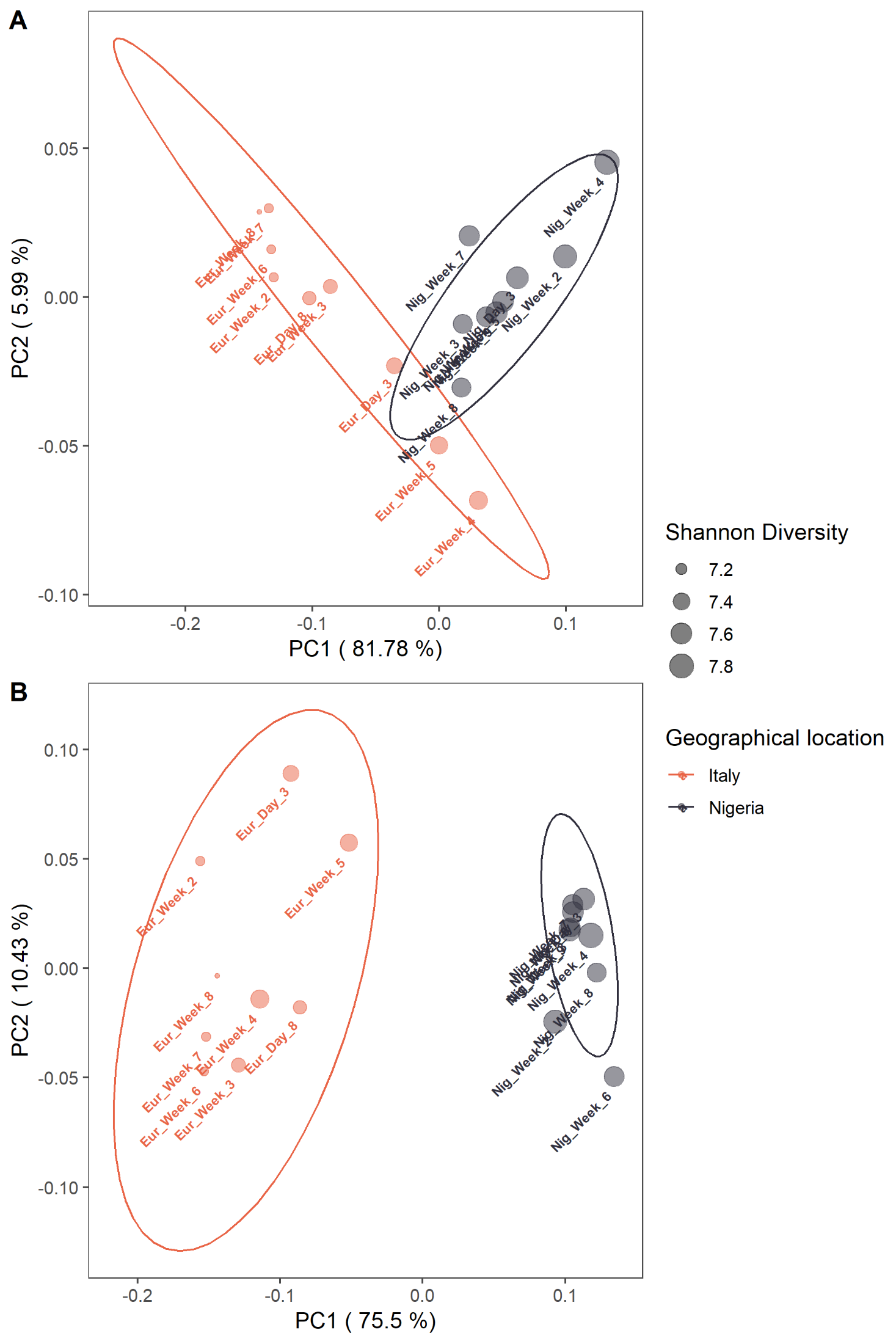


Supplementary Figure 4: The functional structure of an individual’s gut microbiota while in Italy segregates from the microbiome profile associated with life in Nigeria. Principal Coordinates Analysis based on Bray-Curtis (A) and Jaccard (B) distances between the predicted metagenomes associated with short stay in Italy and Nigeria shows significant segregation (p = 0.001, PERMANOVA). Samples were labelled by day or week of collection (see Supplementary Table 1) and coloured by geographical location. Ellipses include 95% confidence area based on the SE of the weighted average of sample coordinates and are coloured by geographical location (Italy: red, Nigeria: black). Dot size is proportional to the Shannon index, as shown in the legend.


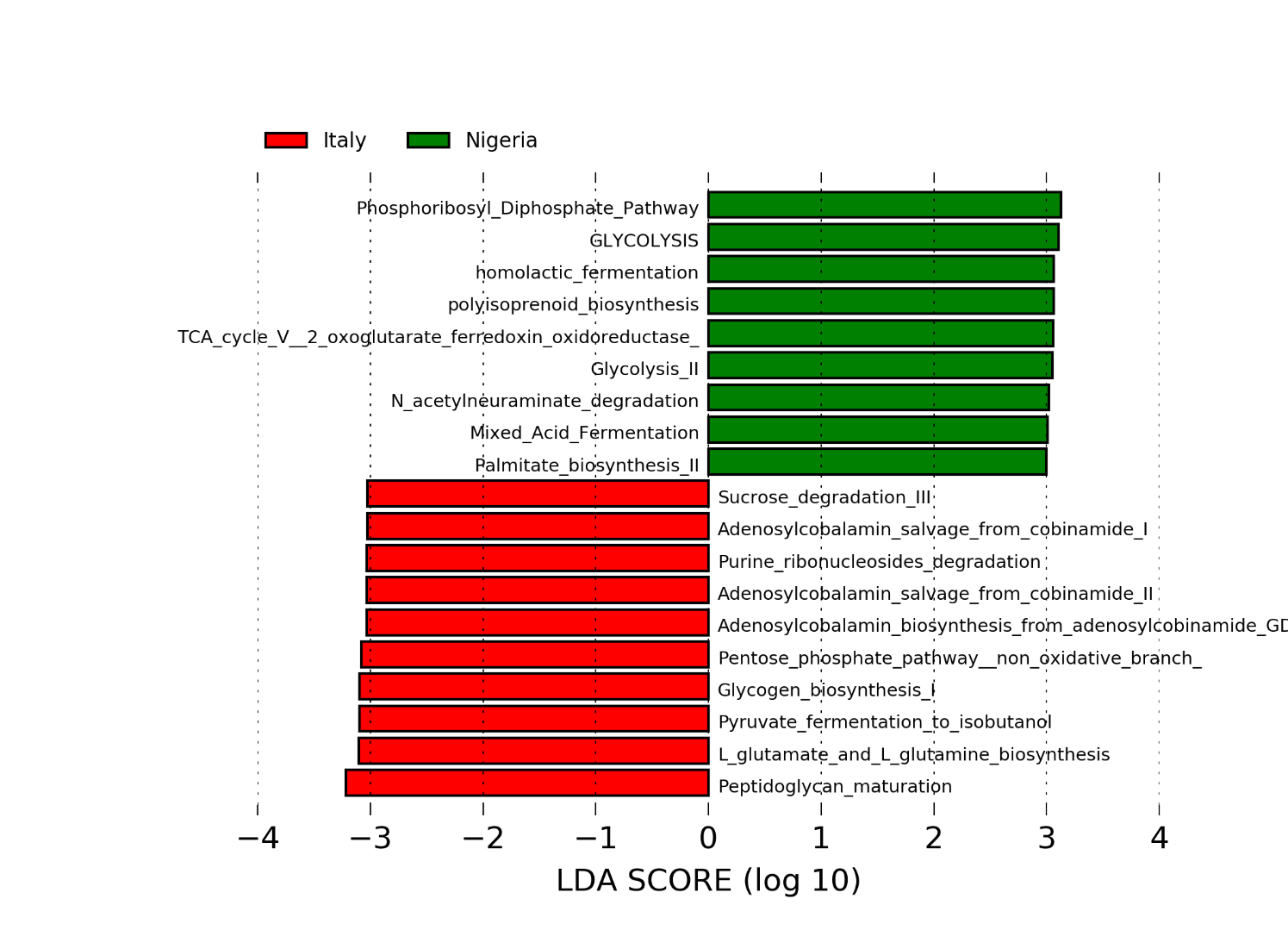


Supplementary Figure 5: Discriminant functional pathways predicted for gut microbiota in an individual on a short stay in Italy and Nigeria. Differentially represented pathways were identified by linear discriminant analysis (LDA) effect size (LEfSe) analysis of PICRUSt-predicted data. The logarithmic threshold for discriminative features was set to 3.0.

**Supplementary Table 1. Timeline of Sample Collection**

| Sample | Timeline of Sample Collection |
| --- | --- |
| F15 (Eur_Day_3) | Sample collected on day 3 after arrival to Italy |
| F20 (Eur_Day_8) | Sample collected on day 8 after arrival to Italy |
| F29 (Eur_Week_2) | Sample collected on the 2nd week after arrival to Italy |
| F2_S86 (Eur_Week_3) | Sample collected on the 3rd week after arrival to Italy |
| F6 (Eur_Week_4) | Sample collected on the 4th week after arrival to Italy |
| F13N (Eur_Week_5) | Sample collected on the 5th week after arrival to Italy |
| F20N (Eur_Week_6) | Sample collected on the 6th week after arrival to Italy |
| F27N (Eur_Week_7) | Sample collected on the 7th week after arrival to Italy |
| F4D (Eur_Week_8) | Sample collected on the 8th week after arrival to Italy |
| F1 (Nig_Day_3) | Sample collected on the 3rd day after return to Nigeria |
| F2_S1 (Nig_Week_2) | Sample collected on the 2^nd^ week   after return to Nigeria |
| F3 (Nig_Week_3) | Sample collected on the 3^rd^ week   after return to Nigeria |
| F4 (Nig_Week_4) | Sample collected on the 4^th^ week   after return to Nigeria |
| F5 (Nig_Week_5) | Sample collected on the 5^th^ week   after return to Nigeria |
| F6 (Nig_Week_6) | Sample collected on the 6^th^ week   after return to Nigeria |
| F7 (Nig_Week_7) | Sample collected on the 7^th^ week   after return to Nigeria |
| F8 (Nig_Week_8) | Sample collected on the 8^th^ week   after return to Nigeria |
| F9 (Nig_Week_9) | Sample collected on the 9^th^ week   after return to Nigeria |
